# Bis(μ-naphthalene-1,8-dicarboxyl­ato)bis­[aqua­(2,2′-bipyridine)zinc(II)] tetra­hydrate

**DOI:** 10.1107/S160053680803643X

**Published:** 2008-11-20

**Authors:** Xia Feng, Yi-Hang Wen

**Affiliations:** a Zhejiang Key Laboratory for Reactive Chemistry on Solid Surfaces, Institute of Physical Chemistry, Zhejiang Normal University, Jinhua, Zhejiang 321004, People’s Republic of China

## Abstract

The title complex, [Zn_2_(C_12_H_6_O_4_)_2_(C_10_H_8_N_2_)_2_(H_2_O)_2_]·4H_2_O, is a binuclear complex with two independent Zn^II^ ions in a slightly disorted trigonal bipyramidal environment, coordinated by one aqua ligand, two naphthalene-1,8-dicarboxyl­ate ligands and one 2,2′-bipyridine ligand. π–π Inter­actions [centroid–centroid distance of 3.8489 (5) Å] and O—H⋯O hydrogen bonds connect the mol­ecules, forming a three-dimensional structure.

## Related literature

1,8-naphthalene­carboxylic anhydride, which is hydrolysed to the naphthalene-1,8-dicarboxyl­ate ligand under hydro­thermal conditions, is employed as a starting material in the preparation of coordination polymers, see: Feng *et al.* (2008 ▶); He *et al.* (2007 ▶); Wen *et al.* (2007 ▶, 2008 ▶). 
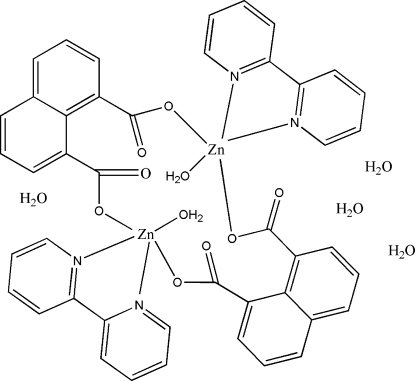


## Experimental

### 

#### Crystal data

[Zn_2_(C_12_H_6_O_4_)_2_(C_10_H_8_N_2_)_2_(H_2_O)_2_]·4H_2_O
*M* *_r_* = 979.54
Triclinic, 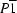

*a* = 10.5774 (12) Å
*b* = 11.3074 (13) Å
*c* = 18.486 (2) Å
α = 83.863 (7)°
β = 80.254 (7)°
γ = 72.197 (6)°
*V* = 2071.2 (4) Å^3^
*Z* = 2
Mo *K*α radiation
μ = 1.24 mm^−1^
*T* = 296 (2) K
0.27 × 0.23 × 0.10 mm

#### Data collection

Bruker APEXII area-detector diffractometer
Absorption correction: multi-scan (*SADABS*; Sheldrick, 1996 ▶) *T*_min_ = 0.72, *T*_max_ = 0.88
34410 measured reflections
9464 independent reflections
6948 reflections with *I* > 2σ(*I*)
*R*_int_ = 0.040

#### Refinement

*R*[*F* ^2^ > 2σ(*F* ^2^)] = 0.040
*wR*(*F* ^2^) = 0.112
*S* = 1.04
9464 reflections
601 parameters
55 restraints
H atoms treated by a mixture of independent and constrained refinement
Δρ_max_ = 0.75 e Å^−3^
Δρ_min_ = −0.61 e Å^−3^

### 

Data collection: *SMART* (Bruker, 2002 ▶); cell refinement: *SAINT* (Bruker, 2002 ▶); data reduction: *SAINT*; program(s) used to solve structure: *SHELXS97* (Sheldrick, 2008 ▶); program(s) used to refine structure: *SHELXL97* (Sheldrick, 2008 ▶); molecular graphics: *SHELXTL* (Sheldrick, 2008 ▶); software used to prepare material for publication: *SHELXTL*.

## Supplementary Material

Crystal structure: contains datablocks I, global. DOI: 10.1107/S160053680803643X/at2661sup1.cif

Structure factors: contains datablocks I. DOI: 10.1107/S160053680803643X/at2661Isup2.hkl

Additional supplementary materials:  crystallographic information; 3D view; checkCIF report

## Figures and Tables

**Table 1:** Hydrogen-bond geometry (Å, °)

*D*—H⋯*A*	*D*—H	H⋯*A*	*D*⋯*A*	*D*—H⋯*A*
O1*W*—H1*WA*⋯O2	0.824 (17)	1.861 (19)	2.644 (3)	158 (3)
O1*W*—H1*WB*⋯O7	0.825 (17)	1.97 (2)	2.763 (3)	162 (3)
O2*W*—H2*WA*⋯O3	0.848 (17)	1.743 (19)	2.570 (3)	164 (3)
O2*W*—H2*WB*⋯O6	0.808 (17)	2.05 (2)	2.778 (3)	150 (3)
O3*W*—H3*WA*⋯O5*W*^i^	0.93 (9)	2.26 (7)	2.807 (7)	117 (6)
O3*W*—H3*WB*⋯O4*W*	0.91 (6)	1.97 (5)	2.785 (6)	149 (7)
O4*W*—H4*WA*⋯O5*W*	0.85	2.16	2.721 (5)	123
O4*W*—H4*WB*⋯O5^ii^	0.85	1.96	2.798 (4)	167
O5*W*—H5*WA*⋯O3*W*^i^	0.85	2.30	2.807 (7)	119
O5*W*—H5*WB*⋯O2	0.85	1.88	2.719 (3)	170
O6*W*—H6*WA*⋯O7	0.829 (18)	2.08 (2)	2.902 (3)	171 (4)
O6*W*—H6*WB*⋯O3^iii^	0.849 (18)	2.117 (19)	2.949 (3)	167 (4)

Symmetry codes: (i) 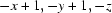; (ii) 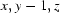; (iii) 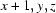.

**Fig. 1.**
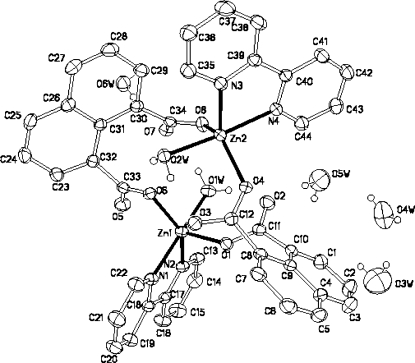
View of the title structure, showing 30% probability displacement ellipsoids.

**Fig. 2.**
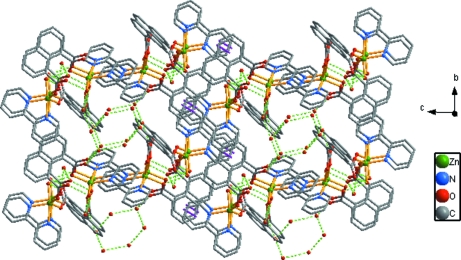
The supramolecular structure of the title complex. All H atoms have been omitted for clarity (the green dash lines represent the O—H···O hydrogen bonds and the red lines represent the π-π interactions).

## supplementary crystallographic information

### Comment

As known, naphthyl-containing aromatic multicarboxylato ligands are versatile building blocks to construct interesting structures with potential properties due to their variety of bridging abilities. In our former work, 1,8-naphthalenecarboxylic anhydride, which is hydrolyzed to naphthalene-1,8-dicarboxylate ligand in hydrothermal condition, is employed as starting material to prepare coordination polymers (Wen *et al.*, 2007; He *et al.*, 2007; Wen *et al.*, 2008; Feng *et al.*, 2008).

The structure of (I) (Fig.1) is a zero-dimensional molecule. Each central Zn^II^ ion is in a slightly distorted trigonal bipyramid environment, coordinated by one aqua ligand, two naphthalene-1,8-dicarboxylate ligands and one 2,2'-bipyridine ligand. Two naphthalene-1,8-dicarboxylate ligands link two five-coordinated Zn^II^ ions to form a eighteen-membered ring. O—H···O hydrogen bonds link independent molecules to form a two-dimensional network. Weak π-π interactions with centroid-centroid distance of 3.849Å connect the layers to yield a three-dimensional structure.

### Experimental

Zn(CH_3_COO)_2_.2H_2_O (0.1108 g, 0.5 mmol), 1, 8-naphthalenecarboxylic anhydride (0.0996 g, 0.5 mmol), NaOH (0.0405 g, 1 mmol), 2,2'-bipy (0.0385 g, 0.25 mmol) and H_2_O-ethanol (2:1, 15 ml) was sealed in a 25 ml stainless-steel reactor with a Teflon liner and was heated at 433 K for 3 d. On completion of the reaction, the reactor was cooled slowly to room temperature and the mixture was filtered, giving pink single crystals suitable for X-ray analysis in yield 39%.

### Refinement

The carbon-bound H-atoms were positioned geometrically and included in the refinement using a riding model [C—H 0.93 Å *U*_iso_(H) = 1.2*U*_eq_(C)]. The water H atoms were located from different maps and their positions were refined isotropically, with O—H distances fixed by O—H = 0.85 (2) Å and H···H = 1.30 (2) Å, their displacement parameters were set to 1.5*U*_eq_(O).

### Crystal data

**Table:**

[Zn_2_(C_12_H_6_O_4_)_2_(C_10_H_8_N_2_)_2_(H_2_O)_2_]·4H_2_O	*Z* = 2
*M**_r_* = 979.54	*F*_000_ = 1008
Triclinic, *P*1	*D*_x_ = 1.571 Mg m^−^^3^
Hall symbol: -P 1	Mo *K*α radiation λ = 0.71073 Å
*a* = 10.5774 (12) Å	Cell parameters from 7692 reflections
*b* = 11.3074 (13) Å	θ = 1.9–27.6º
*c* = 18.486 (2) Å	µ = 1.24 mm^−^^1^
α = 83.863 (7)º	*T* = 296 (2) K
β = 80.254 (7)º	Block, pink
γ = 72.197 (6)º	0.27 × 0.23 × 0.10 mm
*V* = 2071.2 (4) Å^3^	

### Data collection

**Table:**

Bruker APEXII area-detector diffractometer	9464 independent reflections
Radiation source: fine-focus sealed tube	6948 reflections with *I* > 2σ(*I*)
Monochromator: graphite	*R*_int_ = 0.040
*T* = 296(2) K	θ_max_ = 27.6º
ω scans	θ_min_ = 1.9º
Absorption correction: multi-scan(SADABS; Sheldrick, 1996)	*h* = −13→13
*T*_min_ = 0.72, *T*_max_ = 0.88	*k* = −14→14
34410 measured reflections	*l* = −24→24

### Refinement

**Table:**

Refinement on *F*^2^	Secondary atom site location: difference Fourier map
Least-squares matrix: full	Hydrogen site location: inferred from neighbouring sites
*R*[*F*^2^ > 2σ(*F*^2^)] = 0.040	H atoms treated by a mixture of independent and constrained refinement
*wR*(*F*^2^) = 0.112	*w* = 1/[σ^2^(*F*_o_^2^) + (0.0603*P*)^2^ + 0.2947*P*] where *P* = (*F*_o_^2^ + 2*F*_c_^2^)/3
*S* = 1.04	(Δ/σ)_max_ = 0.001
9464 reflections	Δρ_max_ = 0.75 e Å^−^^3^
601 parameters	Δρ_min_ = −0.61 e Å^−^^3^
55 restraints	Extinction correction: none
Primary atom site location: structure-invariant direct methods	

### Special details

**Table:**

Geometry. All e.s.d.'s (except the e.s.d. in the dihedral angle between two l.s. planes) are estimated using the full covariance matrix. The cell e.s.d.'s are taken into account individually in the estimation of e.s.d.'s in distances, angles and torsion angles; correlations between e.s.d.'s in cell parameters are only used when they are defined by crystal symmetry. An approximate (isotropic) treatment of cell e.s.d.'s is used for estimating e.s.d.'s involving l.s. planes.
Refinement. Refinement of *F*^2^ against ALL reflections. The weighted *R*-factor *wR* and goodness of fit *S* are based on *F*^2^, conventional *R*-factors *R* are based on *F*, with *F* set to zero for negative *F*^2^. The threshold expression of *F*^2^ > σ(*F*^2^) is used only for calculating *R*-factors(gt) *etc*. and is not relevant to the choice of reflections for refinement. *R*-factors based on *F*^2^ are statistically about twice as large as those based on *F*, and *R*- factors based on ALL data will be even larger.

### Fractional atomic coordinates and isotropic or equivalent isotropic displacement parameters (Å^2^)

**Table:**

	*x*	*y*	*z*	*U*_iso_*/*U*_eq_	
Zn1	0.19633 (3)	0.96571 (3)	0.149481 (16)	0.03397 (9)	
Zn2	0.20365 (3)	0.75742 (3)	0.377195 (16)	0.03175 (9)	
N1	0.0126 (2)	1.0899 (2)	0.12294 (12)	0.0372 (5)	
N2	0.2418 (2)	1.0038 (2)	0.03553 (12)	0.0365 (5)	
N3	0.1927 (2)	0.7166 (2)	0.49093 (12)	0.0356 (5)	
N4	0.3241 (2)	0.56839 (19)	0.38523 (12)	0.0359 (5)	
O1	0.11681 (17)	0.82126 (16)	0.16625 (10)	0.0368 (4)	
O1W	0.39543 (18)	0.8563 (2)	0.15636 (11)	0.0428 (5)	
H1WA	0.386 (3)	0.786 (2)	0.1646 (18)	0.064*	
H1WB	0.432 (3)	0.876 (3)	0.1873 (16)	0.064*	
O2	0.29945 (19)	0.66321 (19)	0.17988 (12)	0.0497 (5)	
O2W	0.07124 (18)	0.93711 (17)	0.38616 (11)	0.0387 (4)	
H2WA	0.005 (2)	0.929 (3)	0.3695 (17)	0.058*	
H2WB	0.109 (3)	0.979 (3)	0.3572 (15)	0.058*	
O3	−0.09786 (17)	0.88271 (17)	0.32078 (11)	0.0432 (5)	
O3W	0.3790 (6)	0.3502 (6)	−0.0468 (3)	0.1672 (18)	
H3WA	0.437 (8)	0.395 (8)	−0.041 (5)	0.251*	
H3WB	0.377 (9)	0.312 (8)	−0.001 (3)	0.251*	
O4	0.07943 (16)	0.71267 (16)	0.31966 (10)	0.0350 (4)	
O4W	0.4334 (3)	0.2887 (3)	0.0970 (2)	0.1181 (13)	
H4WA	0.4373	0.3198	0.1362	0.177*	
H4WB	0.3754	0.2517	0.1153	0.177*	
O5	0.2689 (2)	1.1513 (2)	0.17454 (11)	0.0517 (5)	
O5W	0.5200 (3)	0.4728 (3)	0.1361 (2)	0.1079 (11)	
H5WA	0.5130	0.5039	0.0925	0.162*	
H5WB	0.4494	0.5267	0.1543	0.162*	
O6	0.17171 (17)	1.03172 (16)	0.25133 (10)	0.0373 (4)	
O6W	0.6463 (2)	1.0749 (2)	0.30347 (15)	0.0595 (6)	
H6WA	0.587 (3)	1.046 (3)	0.296 (2)	0.089*	
H6WB	0.721 (2)	1.019 (3)	0.301 (2)	0.089*	
O7	0.46117 (17)	0.94964 (17)	0.27237 (10)	0.0390 (4)	
O8	0.36064 (17)	0.81452 (16)	0.33168 (11)	0.0402 (4)	
C1	0.1562 (3)	0.5342 (3)	0.11411 (17)	0.0490 (7)	
H1A	0.2444	0.5247	0.0924	0.059*	
C2	0.0848 (4)	0.4619 (3)	0.0920 (2)	0.0635 (9)	
H2A	0.1262	0.4037	0.0569	0.076*	
C3	−0.0445 (4)	0.4768 (3)	0.1220 (2)	0.0606 (9)	
H3A	−0.0923	0.4307	0.1056	0.073*	
C4	−0.1081 (3)	0.5600 (3)	0.17708 (17)	0.0439 (7)	
C5	−0.2442 (3)	0.5775 (3)	0.2066 (2)	0.0528 (8)	
H5A	−0.2914	0.5315	0.1895	0.063*	
C6	−0.3076 (3)	0.6585 (3)	0.25848 (19)	0.0494 (8)	
H6A	−0.3982	0.6705	0.2757	0.059*	
C7	−0.2357 (3)	0.7248 (3)	0.28642 (17)	0.0417 (7)	
H7A	−0.2793	0.7796	0.3231	0.050*	
C8	−0.1022 (2)	0.7105 (2)	0.26084 (15)	0.0344 (6)	
C9	−0.0344 (3)	0.6310 (2)	0.20291 (15)	0.0348 (6)	
C10	0.0992 (3)	0.6185 (2)	0.16696 (15)	0.0359 (6)	
C11	0.1776 (2)	0.7069 (2)	0.17370 (14)	0.0352 (6)	
C12	−0.0337 (2)	0.7753 (2)	0.30211 (14)	0.0328 (5)	
C13	0.3600 (3)	0.9583 (3)	−0.00541 (16)	0.0458 (7)	
H13A	0.4286	0.9014	0.0166	0.055*	
C14	0.3852 (3)	0.9922 (3)	−0.07931 (18)	0.0550 (8)	
H14A	0.4692	0.9592	−0.1064	0.066*	
C15	0.2843 (3)	1.0749 (3)	−0.11157 (18)	0.0608 (9)	
H15A	0.2989	1.0995	−0.1611	0.073*	
C16	0.1611 (3)	1.1218 (3)	−0.07067 (17)	0.0557 (8)	
H16A	0.0913	1.1777	−0.0923	0.067*	
C17	0.1416 (3)	1.0849 (2)	0.00341 (15)	0.0374 (6)	
C18	0.0132 (3)	1.1297 (2)	0.05200 (15)	0.0360 (6)	
C19	−0.1027 (3)	1.2065 (3)	0.02682 (18)	0.0493 (7)	
H19A	−0.1017	1.2335	−0.0225	0.059*	
C20	−0.2188 (3)	1.2422 (3)	0.0757 (2)	0.0560 (8)	
H20A	−0.2971	1.2938	0.0596	0.067*	
C21	−0.2186 (3)	1.2014 (3)	0.1479 (2)	0.0559 (8)	
H21A	−0.2964	1.2250	0.1817	0.067*	
C22	−0.1012 (3)	1.1249 (3)	0.16987 (17)	0.0478 (7)	
H22A	−0.1011	1.0967	0.2190	0.057*	
C23	0.1569 (3)	1.3201 (3)	0.29420 (18)	0.0451 (7)	
H23A	0.1422	1.3569	0.2477	0.054*	
C24	0.1184 (3)	1.3945 (3)	0.3548 (2)	0.0530 (8)	
H24A	0.0794	1.4799	0.3485	0.064*	
C25	0.1385 (3)	1.3411 (3)	0.42265 (19)	0.0495 (7)	
H25A	0.1072	1.3896	0.4632	0.059*	
C26	0.2062 (3)	1.2129 (3)	0.43301 (16)	0.0409 (6)	
C27	0.2336 (3)	1.1603 (3)	0.50399 (17)	0.0509 (8)	
H27A	0.2045	1.2104	0.5439	0.061*	
C28	0.3013 (3)	1.0385 (3)	0.51466 (17)	0.0536 (8)	
H28A	0.3178	1.0052	0.5615	0.064*	
C29	0.3461 (3)	0.9633 (3)	0.45468 (16)	0.0452 (7)	
H29A	0.3936	0.8800	0.4623	0.054*	
C30	0.3222 (2)	1.0086 (2)	0.38493 (14)	0.0335 (6)	
C31	0.2498 (2)	1.1365 (2)	0.37202 (14)	0.0322 (5)	
C32	0.2158 (2)	1.1942 (2)	0.30217 (15)	0.0353 (6)	
C33	0.2235 (3)	1.1223 (2)	0.23787 (15)	0.0367 (6)	
C34	0.3838 (2)	0.9198 (2)	0.32488 (15)	0.0335 (6)	
C35	0.1273 (3)	0.7988 (3)	0.54229 (16)	0.0432 (7)	
H35A	0.0798	0.8787	0.5270	0.052*	
C36	0.1279 (3)	0.7698 (3)	0.61537 (17)	0.0483 (7)	
H36A	0.0839	0.8295	0.6492	0.058*	
C37	0.1941 (3)	0.6512 (3)	0.63862 (17)	0.0562 (8)	
H37A	0.1939	0.6291	0.6885	0.067*	
C38	0.2604 (3)	0.5660 (3)	0.58776 (17)	0.0495 (7)	
H38A	0.3055	0.4851	0.6028	0.059*	
C39	0.2603 (2)	0.6008 (2)	0.51321 (15)	0.0348 (6)	
C40	0.3340 (2)	0.5173 (2)	0.45381 (15)	0.0340 (6)	
C41	0.4099 (3)	0.3947 (3)	0.46601 (18)	0.0464 (7)	
H41A	0.4175	0.3609	0.5138	0.056*	
C42	0.4735 (3)	0.3240 (3)	0.40738 (19)	0.0521 (8)	
H42A	0.5239	0.2416	0.4149	0.062*	
C43	0.4618 (3)	0.3763 (3)	0.33749 (19)	0.0515 (8)	
H43A	0.5038	0.3300	0.2968	0.062*	
C44	0.3867 (3)	0.4988 (3)	0.32860 (17)	0.0459 (7)	
H44A	0.3795	0.5344	0.2812	0.055*	

### Atomic displacement parameters (Å^2^)

**Table:**

	*U*^11^	*U*^22^	*U*^33^	*U*^12^	*U*^13^	*U*^23^
Zn1	0.03452 (16)	0.03960 (18)	0.02821 (17)	−0.01121 (13)	−0.00770 (12)	0.00229 (13)
Zn2	0.03274 (16)	0.02909 (16)	0.03286 (18)	−0.00729 (12)	−0.00830 (12)	0.00109 (12)
N1	0.0382 (12)	0.0408 (13)	0.0336 (12)	−0.0122 (10)	−0.0093 (10)	0.0020 (10)
N2	0.0388 (12)	0.0432 (13)	0.0303 (12)	−0.0155 (10)	−0.0079 (10)	0.0010 (10)
N3	0.0353 (11)	0.0327 (12)	0.0380 (13)	−0.0085 (9)	−0.0080 (10)	0.0016 (10)
N4	0.0381 (11)	0.0299 (11)	0.0391 (13)	−0.0065 (9)	−0.0109 (10)	−0.0009 (10)
O1	0.0363 (9)	0.0335 (10)	0.0412 (11)	−0.0107 (8)	−0.0095 (8)	0.0030 (8)
O1W	0.0355 (8)	0.0534 (13)	0.0393 (11)	−0.0132 (8)	−0.0079 (8)	0.0024 (10)
O2	0.0385 (10)	0.0480 (12)	0.0596 (14)	−0.0069 (9)	−0.0121 (10)	0.0004 (10)
O2W	0.0365 (10)	0.0325 (10)	0.0477 (12)	−0.0085 (8)	−0.0102 (9)	−0.0033 (9)
O3	0.0361 (10)	0.0396 (11)	0.0524 (12)	−0.0026 (8)	−0.0126 (9)	−0.0116 (9)
O3W	0.183 (5)	0.195 (5)	0.125 (4)	−0.062 (4)	−0.011 (4)	−0.009 (3)
O4	0.0342 (9)	0.0324 (9)	0.0385 (10)	−0.0048 (7)	−0.0145 (8)	−0.0022 (8)
O4W	0.084 (2)	0.083 (2)	0.173 (4)	−0.0234 (17)	0.019 (2)	−0.006 (2)
O5	0.0615 (13)	0.0598 (13)	0.0341 (12)	−0.0205 (11)	−0.0081 (10)	0.0063 (10)
O5W	0.0714 (18)	0.102 (2)	0.136 (3)	0.0045 (17)	−0.0108 (19)	−0.041 (2)
O6	0.0417 (10)	0.0381 (10)	0.0366 (10)	−0.0146 (8)	−0.0137 (8)	−0.0001 (8)
O6W	0.0530 (13)	0.0523 (14)	0.0749 (16)	−0.0155 (10)	−0.0125 (13)	−0.0058 (12)
O7	0.0333 (9)	0.0439 (11)	0.0408 (11)	−0.0142 (8)	−0.0051 (8)	0.0013 (9)
O8	0.0349 (9)	0.0324 (10)	0.0516 (12)	−0.0097 (8)	−0.0023 (8)	−0.0014 (9)
C1	0.0535 (17)	0.0436 (17)	0.0477 (18)	−0.0086 (14)	−0.0102 (15)	−0.0045 (14)
C2	0.087 (3)	0.049 (2)	0.058 (2)	−0.0159 (18)	−0.0169 (19)	−0.0201 (17)
C3	0.078 (2)	0.053 (2)	0.063 (2)	−0.0280 (17)	−0.0266 (19)	−0.0073 (17)
C4	0.0553 (17)	0.0394 (16)	0.0446 (17)	−0.0183 (13)	−0.0231 (14)	0.0034 (13)
C5	0.0549 (18)	0.0497 (18)	0.067 (2)	−0.0287 (15)	−0.0309 (17)	0.0120 (17)
C6	0.0389 (15)	0.0492 (18)	0.064 (2)	−0.0175 (13)	−0.0170 (15)	0.0111 (16)
C7	0.0363 (14)	0.0406 (15)	0.0477 (17)	−0.0095 (12)	−0.0132 (13)	0.0060 (13)
C8	0.0362 (13)	0.0333 (14)	0.0352 (15)	−0.0103 (11)	−0.0141 (11)	0.0061 (11)
C9	0.0409 (14)	0.0305 (13)	0.0363 (15)	−0.0109 (11)	−0.0178 (12)	0.0045 (11)
C10	0.0426 (14)	0.0284 (13)	0.0349 (15)	−0.0052 (11)	−0.0125 (12)	0.0017 (11)
C11	0.0346 (13)	0.0407 (15)	0.0281 (14)	−0.0077 (11)	−0.0056 (11)	−0.0011 (11)
C12	0.0340 (13)	0.0354 (14)	0.0299 (14)	−0.0127 (11)	−0.0045 (11)	0.0010 (11)
C13	0.0402 (15)	0.0597 (19)	0.0399 (17)	−0.0185 (13)	−0.0049 (13)	−0.0036 (14)
C14	0.0535 (18)	0.069 (2)	0.0431 (18)	−0.0258 (16)	0.0070 (15)	−0.0055 (16)
C15	0.068 (2)	0.079 (2)	0.0332 (17)	−0.0253 (19)	−0.0030 (16)	0.0110 (16)
C16	0.061 (2)	0.068 (2)	0.0358 (17)	−0.0178 (17)	−0.0149 (15)	0.0126 (16)
C17	0.0471 (15)	0.0385 (15)	0.0319 (14)	−0.0182 (12)	−0.0127 (12)	0.0025 (12)
C18	0.0410 (14)	0.0347 (14)	0.0355 (15)	−0.0135 (11)	−0.0132 (12)	0.0037 (11)
C19	0.0533 (18)	0.0475 (17)	0.0455 (18)	−0.0095 (14)	−0.0184 (15)	0.0060 (14)
C20	0.0463 (17)	0.0517 (19)	0.067 (2)	−0.0051 (14)	−0.0224 (17)	0.0040 (17)
C21	0.0388 (16)	0.060 (2)	0.063 (2)	−0.0062 (14)	−0.0051 (15)	−0.0041 (17)
C22	0.0438 (16)	0.0539 (18)	0.0427 (17)	−0.0121 (13)	−0.0059 (13)	0.0027 (14)
C23	0.0510 (16)	0.0367 (15)	0.0507 (18)	−0.0151 (13)	−0.0160 (14)	0.0046 (13)
C24	0.0558 (18)	0.0318 (15)	0.075 (2)	−0.0122 (13)	−0.0204 (17)	−0.0036 (15)
C25	0.0515 (17)	0.0433 (17)	0.058 (2)	−0.0157 (14)	−0.0076 (15)	−0.0181 (15)
C26	0.0410 (14)	0.0456 (16)	0.0425 (17)	−0.0202 (12)	−0.0081 (12)	−0.0044 (13)
C27	0.0605 (19)	0.062 (2)	0.0379 (17)	−0.0275 (16)	−0.0056 (14)	−0.0110 (15)
C28	0.068 (2)	0.064 (2)	0.0357 (17)	−0.0280 (17)	−0.0161 (15)	0.0076 (15)
C29	0.0516 (16)	0.0448 (17)	0.0425 (17)	−0.0170 (13)	−0.0156 (14)	0.0061 (14)
C30	0.0326 (12)	0.0371 (14)	0.0352 (15)	−0.0163 (11)	−0.0073 (11)	0.0010 (11)
C31	0.0329 (12)	0.0343 (14)	0.0335 (14)	−0.0151 (10)	−0.0073 (11)	−0.0006 (11)
C32	0.0371 (13)	0.0340 (14)	0.0383 (15)	−0.0148 (11)	−0.0083 (11)	0.0009 (12)
C33	0.0376 (13)	0.0361 (14)	0.0354 (15)	−0.0072 (11)	−0.0136 (12)	0.0052 (12)
C34	0.0262 (12)	0.0358 (14)	0.0391 (15)	−0.0074 (10)	−0.0132 (11)	0.0034 (12)
C35	0.0416 (15)	0.0417 (16)	0.0438 (17)	−0.0087 (12)	−0.0062 (13)	−0.0017 (13)
C36	0.0503 (17)	0.0539 (19)	0.0360 (17)	−0.0089 (14)	−0.0029 (13)	−0.0069 (14)
C37	0.065 (2)	0.068 (2)	0.0299 (16)	−0.0133 (17)	−0.0075 (15)	0.0041 (15)
C38	0.0579 (18)	0.0446 (17)	0.0416 (18)	−0.0085 (14)	−0.0150 (15)	0.0093 (14)
C39	0.0315 (12)	0.0370 (14)	0.0385 (15)	−0.0130 (11)	−0.0102 (11)	0.0040 (12)
C40	0.0328 (13)	0.0324 (13)	0.0403 (15)	−0.0124 (10)	−0.0115 (11)	0.0013 (11)
C41	0.0460 (16)	0.0370 (15)	0.0537 (19)	−0.0062 (12)	−0.0168 (14)	0.0053 (14)
C42	0.0492 (17)	0.0354 (16)	0.067 (2)	−0.0026 (13)	−0.0163 (16)	−0.0010 (15)
C43	0.0493 (17)	0.0425 (17)	0.056 (2)	−0.0018 (13)	−0.0061 (15)	−0.0129 (15)
C44	0.0525 (17)	0.0423 (16)	0.0398 (17)	−0.0086 (13)	−0.0077 (14)	−0.0027 (13)

### Geometric parameters (Å, °)

**Table:**

Zn1—O1	2.0285 (17)	C9—C10	1.428 (4)
Zn1—O6	2.0458 (19)	C10—C11	1.506 (4)
Zn1—N2	2.108 (2)	C13—C14	1.382 (4)
Zn1—O1W	2.1085 (19)	C13—H13A	0.9300
Zn1—N1	2.121 (2)	C14—C15	1.363 (5)
Zn2—O8	1.9871 (17)	C14—H14A	0.9300
Zn2—O4	2.0270 (17)	C15—C16	1.371 (5)
Zn2—O2W	2.0906 (19)	C15—H15A	0.9300
Zn2—N3	2.095 (2)	C16—C17	1.388 (4)
Zn2—N4	2.132 (2)	C16—H16A	0.9300
N1—C22	1.337 (4)	C17—C18	1.474 (4)
N1—C18	1.340 (3)	C18—C19	1.389 (4)
N2—C13	1.330 (4)	C19—C20	1.373 (5)
N2—C17	1.349 (3)	C19—H19A	0.9300
N3—C39	1.344 (3)	C20—C21	1.366 (5)
N3—C35	1.348 (4)	C20—H20A	0.9300
N4—C44	1.333 (4)	C21—C22	1.376 (4)
N4—C40	1.343 (3)	C21—H21A	0.9300
O1—C11	1.261 (3)	C22—H22A	0.9300
O1W—H1WA	0.824 (17)	C23—C32	1.372 (4)
O1W—H1WB	0.825 (17)	C23—C24	1.403 (4)
O2—C11	1.251 (3)	C23—H23A	0.9300
O2W—H2WA	0.848 (17)	C24—C25	1.353 (5)
O2W—H2WB	0.808 (17)	C24—H24A	0.9300
O3—C12	1.249 (3)	C25—C26	1.416 (4)
O3W—H3WA	0.93 (9)	C25—H25A	0.9300
O3W—H3WB	0.91 (6)	C26—C27	1.420 (4)
O4—C12	1.266 (3)	C26—C31	1.421 (4)
O4W—H4WA	0.8502	C27—C28	1.354 (5)
O4W—H4WB	0.8504	C27—H27A	0.9300
O5—C33	1.238 (3)	C28—C29	1.398 (4)
O5W—H5WA	0.8503	C28—H28A	0.9300
O5W—H5WB	0.8499	C29—C30	1.371 (4)
O6—C33	1.286 (3)	C29—H29A	0.9300
O6W—H6WA	0.829 (18)	C30—C31	1.430 (3)
O6W—H6WB	0.849 (18)	C30—C34	1.503 (4)
O7—C34	1.247 (3)	C31—C32	1.434 (4)
O8—C34	1.276 (3)	C32—C33	1.488 (4)
C1—C10	1.370 (4)	C35—C36	1.357 (4)
C1—C2	1.399 (4)	C35—H35A	0.9300
C1—H1A	0.9300	C36—C37	1.370 (4)
C2—C3	1.353 (5)	C36—H36A	0.9300
C2—H2A	0.9300	C37—C38	1.365 (4)
C3—C4	1.401 (5)	C37—H37A	0.9300
C3—H3A	0.9300	C38—C39	1.392 (4)
C4—C5	1.411 (4)	C38—H38A	0.9300
C4—C9	1.440 (4)	C39—C40	1.479 (4)
C5—C6	1.343 (5)	C40—C41	1.390 (4)
C5—H5A	0.9300	C41—C42	1.370 (4)
C6—C7	1.404 (4)	C41—H41A	0.9300
C6—H6A	0.9300	C42—C43	1.371 (4)
C7—C8	1.376 (4)	C42—H42A	0.9300
C7—H7A	0.9300	C43—C44	1.378 (4)
C8—C9	1.422 (4)	C43—H43A	0.9300
C8—C12	1.508 (4)	C44—H44A	0.9300
			
O1—Zn1—O6	104.86 (7)	C14—C15—H15A	120.1
O1—Zn1—N2	109.17 (8)	C16—C15—H15A	120.1
O6—Zn1—N2	144.94 (8)	C15—C16—C17	119.3 (3)
O1—Zn1—O1W	95.57 (7)	C15—C16—H16A	120.4
O6—Zn1—O1W	93.01 (8)	C17—C16—H16A	120.4
N2—Zn1—O1W	91.91 (8)	N2—C17—C16	121.0 (3)
O1—Zn1—N1	91.35 (8)	N2—C17—C18	115.6 (2)
O6—Zn1—N1	93.69 (8)	C16—C17—C18	123.4 (3)
N2—Zn1—N1	77.50 (8)	N1—C18—C19	120.8 (3)
O1W—Zn1—N1	168.82 (8)	N1—C18—C17	116.1 (2)
O8—Zn2—O4	124.30 (8)	C19—C18—C17	123.0 (3)
O8—Zn2—O2W	94.47 (7)	C20—C19—C18	119.2 (3)
O4—Zn2—O2W	89.84 (7)	C20—C19—H19A	120.4
O8—Zn2—N3	114.43 (8)	C18—C19—H19A	120.4
O4—Zn2—N3	120.50 (8)	C21—C20—C19	119.6 (3)
O2W—Zn2—N3	94.63 (8)	C21—C20—H20A	120.2
O8—Zn2—N4	92.17 (8)	C19—C20—H20A	120.2
O4—Zn2—N4	91.54 (8)	C20—C21—C22	118.8 (3)
O2W—Zn2—N4	170.91 (8)	C20—C21—H21A	120.6
N3—Zn2—N4	76.94 (9)	C22—C21—H21A	120.6
C22—N1—C18	119.3 (2)	N1—C22—C21	122.2 (3)
C22—N1—Zn1	125.40 (19)	N1—C22—H22A	118.9
C18—N1—Zn1	115.17 (18)	C21—C22—H22A	118.9
C13—N2—C17	118.7 (2)	C32—C23—C24	121.3 (3)
C13—N2—Zn1	125.72 (19)	C32—C23—H23A	119.4
C17—N2—Zn1	115.56 (18)	C24—C23—H23A	119.4
C39—N3—C35	118.5 (2)	C25—C24—C23	119.5 (3)
C39—N3—Zn2	116.67 (18)	C25—C24—H24A	120.2
C35—N3—Zn2	124.75 (18)	C23—C24—H24A	120.2
C44—N4—C40	118.9 (2)	C24—C25—C26	121.2 (3)
C44—N4—Zn2	125.46 (19)	C24—C25—H25A	119.4
C40—N4—Zn2	115.66 (18)	C26—C25—H25A	119.4
C11—O1—Zn1	127.96 (16)	C25—C26—C27	120.3 (3)
Zn1—O1W—H1WA	102 (2)	C25—C26—C31	120.1 (3)
Zn1—O1W—H1WB	116 (2)	C27—C26—C31	119.6 (3)
H1WA—O1W—H1WB	113 (3)	C28—C27—C26	121.2 (3)
Zn2—O2W—H2WA	101 (2)	C28—C27—H27A	119.4
Zn2—O2W—H2WB	103 (2)	C26—C27—H27A	119.4
H2WA—O2W—H2WB	110 (2)	C27—C28—C29	119.5 (3)
H3WA—O3W—H3WB	97 (8)	C27—C28—H28A	120.2
C12—O4—Zn2	131.02 (16)	C29—C28—H28A	120.2
H4WA—O4W—H4WB	97.3	C30—C29—C28	121.9 (3)
H5WA—O5W—H5WB	93.0	C30—C29—H29A	119.0
C33—O6—Zn1	102.49 (16)	C28—C29—H29A	119.0
H6WA—O6W—H6WB	110 (3)	C29—C30—C31	119.9 (3)
C34—O8—Zn2	133.57 (17)	C29—C30—C34	116.3 (2)
C10—C1—C2	121.6 (3)	C31—C30—C34	123.7 (2)
C10—C1—H1A	119.2	C26—C31—C30	117.9 (2)
C2—C1—H1A	119.2	C26—C31—C32	116.9 (2)
C3—C2—C1	119.8 (3)	C30—C31—C32	125.1 (2)
C3—C2—H2A	120.1	C23—C32—C31	120.6 (3)
C1—C2—H2A	120.1	C23—C32—C33	115.7 (2)
C2—C3—C4	121.5 (3)	C31—C32—C33	123.1 (2)
C2—C3—H3A	119.2	O5—C33—O6	121.4 (3)
C4—C3—H3A	119.2	O5—C33—C32	122.9 (2)
C3—C4—C5	121.1 (3)	O6—C33—C32	115.6 (2)
C3—C4—C9	119.5 (3)	O7—C34—O8	122.8 (3)
C5—C4—C9	119.4 (3)	O7—C34—C30	118.4 (2)
C6—C5—C4	122.0 (3)	O8—C34—C30	118.7 (2)
C6—C5—H5A	119.0	N3—C35—C36	122.7 (3)
C4—C5—H5A	119.0	N3—C35—H35A	118.6
C5—C6—C7	119.3 (3)	C36—C35—H35A	118.6
C5—C6—H6A	120.3	C35—C36—C37	119.1 (3)
C7—C6—H6A	120.3	C35—C36—H36A	120.4
C8—C7—C6	121.5 (3)	C37—C36—H36A	120.4
C8—C7—H7A	119.2	C38—C37—C36	119.3 (3)
C6—C7—H7A	119.2	C38—C37—H37A	120.3
C7—C8—C9	120.4 (2)	C36—C37—H37A	120.3
C7—C8—C12	115.7 (3)	C37—C38—C39	119.6 (3)
C9—C8—C12	123.7 (2)	C37—C38—H38A	120.2
C8—C9—C10	125.4 (2)	C39—C38—H38A	120.2
C8—C9—C4	117.1 (2)	N3—C39—C38	120.7 (3)
C10—C9—C4	117.4 (3)	N3—C39—C40	115.5 (2)
C1—C10—C9	120.0 (3)	C38—C39—C40	123.8 (2)
C1—C10—C11	115.1 (2)	N4—C40—C41	120.9 (3)
C9—C10—C11	124.2 (2)	N4—C40—C39	115.2 (2)
O2—C11—O1	124.6 (2)	C41—C40—C39	123.9 (3)
O2—C11—C10	118.8 (2)	C42—C41—C40	119.7 (3)
O1—C11—C10	116.3 (2)	C42—C41—H41A	120.2
O3—C12—O4	125.6 (2)	C40—C41—H41A	120.2
O3—C12—C8	117.1 (2)	C41—C42—C43	119.1 (3)
O4—C12—C8	117.1 (2)	C41—C42—H42A	120.5
N2—C13—C14	122.8 (3)	C43—C42—H42A	120.5
N2—C13—H13A	118.6	C42—C43—C44	118.8 (3)
C14—C13—H13A	118.6	C42—C43—H43A	120.6
C15—C14—C13	118.5 (3)	C44—C43—H43A	120.6
C15—C14—H14A	120.8	N4—C44—C43	122.7 (3)
C13—C14—H14A	120.8	N4—C44—H44A	118.7
C14—C15—C16	119.8 (3)	C43—C44—H44A	118.7
			
O1—Zn1—N1—C22	67.5 (2)	C13—C14—C15—C16	−0.4 (5)
O6—Zn1—N1—C22	−37.5 (2)	C14—C15—C16—C17	0.7 (5)
N2—Zn1—N1—C22	176.9 (2)	C13—N2—C17—C16	−0.7 (4)
O1W—Zn1—N1—C22	−164.2 (4)	Zn1—N2—C17—C16	177.1 (2)
O1—Zn1—N1—C18	−109.18 (19)	C13—N2—C17—C18	179.1 (2)
O6—Zn1—N1—C18	145.83 (18)	Zn1—N2—C17—C18	−3.1 (3)
N2—Zn1—N1—C18	0.16 (18)	C15—C16—C17—N2	−0.1 (5)
O1W—Zn1—N1—C18	19.1 (5)	C15—C16—C17—C18	−180.0 (3)
O1—Zn1—N2—C13	−93.7 (2)	C22—N1—C18—C19	−0.1 (4)
O6—Zn1—N2—C13	100.9 (2)	Zn1—N1—C18—C19	176.8 (2)
O1W—Zn1—N2—C13	2.9 (2)	C22—N1—C18—C17	−178.7 (2)
N1—Zn1—N2—C13	179.3 (2)	Zn1—N1—C18—C17	−1.8 (3)
O1—Zn1—N2—C17	88.69 (19)	N2—C17—C18—N1	3.3 (3)
O6—Zn1—N2—C17	−76.8 (2)	C16—C17—C18—N1	−176.9 (3)
O1W—Zn1—N2—C17	−174.74 (18)	N2—C17—C18—C19	−175.3 (3)
N1—Zn1—N2—C17	1.65 (18)	C16—C17—C18—C19	4.5 (4)
O8—Zn2—N3—C39	−86.63 (18)	N1—C18—C19—C20	0.3 (4)
O4—Zn2—N3—C39	83.77 (18)	C17—C18—C19—C20	178.8 (3)
O2W—Zn2—N3—C39	176.33 (17)	C18—C19—C20—C21	−0.2 (5)
N4—Zn2—N3—C39	−0.22 (16)	C19—C20—C21—C22	−0.2 (5)
O8—Zn2—N3—C35	91.0 (2)	C18—N1—C22—C21	−0.3 (4)
O4—Zn2—N3—C35	−98.6 (2)	Zn1—N1—C22—C21	−176.8 (2)
O2W—Zn2—N3—C35	−6.1 (2)	C20—C21—C22—N1	0.4 (5)
N4—Zn2—N3—C35	177.4 (2)	C32—C23—C24—C25	−0.7 (4)
O8—Zn2—N4—C44	−66.1 (2)	C23—C24—C25—C26	4.5 (4)
O4—Zn2—N4—C44	58.3 (2)	C24—C25—C26—C27	176.1 (3)
N3—Zn2—N4—C44	179.3 (2)	C24—C25—C26—C31	−2.6 (4)
O8—Zn2—N4—C40	114.95 (18)	C25—C26—C27—C28	−178.3 (3)
O4—Zn2—N4—C40	−120.64 (17)	C31—C26—C27—C28	0.4 (4)
N3—Zn2—N4—C40	0.36 (17)	C26—C27—C28—C29	0.6 (5)
O6—Zn1—O1—C11	−97.8 (2)	C27—C28—C29—C30	−0.9 (5)
N2—Zn1—O1—C11	90.8 (2)	C28—C29—C30—C31	0.3 (4)
O1W—Zn1—O1—C11	−3.2 (2)	C28—C29—C30—C34	176.1 (2)
N1—Zn1—O1—C11	168.0 (2)	C25—C26—C31—C30	177.7 (2)
O8—Zn2—O4—C12	−97.7 (2)	C27—C26—C31—C30	−0.9 (3)
O2W—Zn2—O4—C12	−2.4 (2)	C25—C26—C31—C32	−3.1 (3)
N3—Zn2—O4—C12	92.9 (2)	C27—C26—C31—C32	178.3 (2)
N4—Zn2—O4—C12	168.6 (2)	C29—C30—C31—C26	0.6 (3)
O1—Zn1—O6—C33	179.91 (15)	C34—C30—C31—C26	−174.9 (2)
N2—Zn1—O6—C33	−14.3 (2)	C29—C30—C31—C32	−178.5 (2)
O1W—Zn1—O6—C33	83.34 (16)	C34—C30—C31—C32	6.0 (4)
N1—Zn1—O6—C33	−87.71 (16)	C24—C23—C32—C31	−5.2 (4)
O4—Zn2—O8—C34	106.8 (2)	C24—C23—C32—C33	166.7 (3)
O2W—Zn2—O8—C34	13.9 (2)	C26—C31—C32—C23	6.9 (3)
N3—Zn2—O8—C34	−83.2 (2)	C30—C31—C32—C23	−174.0 (2)
N4—Zn2—O8—C34	−159.9 (2)	C26—C31—C32—C33	−164.3 (2)
C10—C1—C2—C3	1.5 (5)	C30—C31—C32—C33	14.8 (4)
C1—C2—C3—C4	−2.5 (5)	Zn1—O6—C33—O5	1.8 (3)
C2—C3—C4—C5	178.2 (3)	Zn1—O6—C33—C32	177.63 (17)
C2—C3—C4—C9	−0.3 (5)	C23—C32—C33—O5	50.6 (4)
C3—C4—C5—C6	−178.8 (3)	C31—C32—C33—O5	−137.8 (3)
C9—C4—C5—C6	−0.2 (4)	C23—C32—C33—O6	−125.2 (3)
C4—C5—C6—C7	−2.4 (5)	C31—C32—C33—O6	46.4 (3)
C5—C6—C7—C8	1.3 (4)	Zn2—O8—C34—O7	−150.56 (19)
C6—C7—C8—C9	2.4 (4)	Zn2—O8—C34—C30	33.3 (3)
C6—C7—C8—C12	−172.8 (2)	C29—C30—C34—O7	−122.4 (3)
C7—C8—C9—C10	173.3 (2)	C31—C30—C34—O7	53.3 (3)
C12—C8—C9—C10	−11.9 (4)	C29—C30—C34—O8	53.9 (3)
C7—C8—C9—C4	−4.8 (4)	C31—C30—C34—O8	−130.5 (2)
C12—C8—C9—C4	170.0 (2)	C39—N3—C35—C36	0.6 (4)
C3—C4—C9—C8	−177.6 (3)	Zn2—N3—C35—C36	−177.0 (2)
C5—C4—C9—C8	3.8 (4)	N3—C35—C36—C37	−2.0 (5)
C3—C4—C9—C10	4.1 (4)	C35—C36—C37—C38	1.5 (5)
C5—C4—C9—C10	−174.5 (2)	C36—C37—C38—C39	0.3 (5)
C2—C1—C10—C9	2.4 (4)	C35—N3—C39—C38	1.2 (4)
C2—C1—C10—C11	−168.1 (3)	Zn2—N3—C39—C38	179.0 (2)
C8—C9—C10—C1	176.8 (3)	C35—N3—C39—C40	−177.7 (2)
C4—C9—C10—C1	−5.0 (4)	Zn2—N3—C39—C40	0.1 (3)
C8—C9—C10—C11	−13.7 (4)	C37—C38—C39—N3	−1.7 (4)
C4—C9—C10—C11	164.5 (2)	C37—C38—C39—C40	177.2 (3)
Zn1—O1—C11—O2	8.2 (4)	C44—N4—C40—C41	1.1 (4)
Zn1—O1—C11—C10	−165.51 (17)	Zn2—N4—C40—C41	−179.89 (19)
C1—C10—C11—O2	−51.0 (3)	C44—N4—C40—C39	−179.4 (2)
C9—C10—C11—O2	139.0 (3)	Zn2—N4—C40—C39	−0.4 (3)
C1—C10—C11—O1	123.1 (3)	N3—C39—C40—N4	0.2 (3)
C9—C10—C11—O1	−46.9 (4)	C38—C39—C40—N4	−178.7 (2)
Zn2—O4—C12—O3	−3.4 (4)	N3—C39—C40—C41	179.7 (2)
Zn2—O4—C12—C8	−177.67 (16)	C38—C39—C40—C41	0.8 (4)
C7—C8—C12—O3	−41.7 (3)	N4—C40—C41—C42	−1.3 (4)
C9—C8—C12—O3	143.2 (3)	C39—C40—C41—C42	179.3 (3)
C7—C8—C12—O4	133.0 (3)	C40—C41—C42—C43	0.6 (4)
C9—C8—C12—O4	−42.0 (3)	C41—C42—C43—C44	0.3 (5)
C17—N2—C13—C14	1.0 (4)	C40—N4—C44—C43	−0.2 (4)
Zn1—N2—C13—C14	−176.5 (2)	Zn2—N4—C44—C43	−179.1 (2)
N2—C13—C14—C15	−0.5 (5)	C42—C43—C44—N4	−0.5 (5)

### Hydrogen-bond geometry (Å, °)

**Table:**

*D*—H···*A*	*D*—H	H···*A*	*D*···*A*	*D*—H···*A*
O1W—H1WA···O2	0.824 (17)	1.861 (19)	2.644 (3)	158 (3)
O1W—H1WB···O7	0.825 (17)	1.97 (2)	2.763 (3)	162 (3)
O2W—H2WA···O3	0.848 (17)	1.743 (19)	2.570 (3)	164 (3)
O2W—H2WB···O6	0.808 (17)	2.05 (2)	2.778 (3)	150 (3)
O3W—H3WA···O5W^i^	0.93 (9)	2.26 (7)	2.807 (7)	117 (6)
O3W—H3WB···O4W	0.91 (6)	1.97 (5)	2.785 (6)	149 (7)
O4W—H4WA···O5W	0.85	2.16	2.721 (5)	123
O4W—H4WB···O5^ii^	0.85	1.96	2.798 (4)	167
O5W—H5WA···O3W^i^	0.85	2.30	2.807 (7)	119
O5W—H5WB···O2	0.85	1.88	2.719 (3)	170
O6W—H6WA···O7	0.829 (18)	2.08 (2)	2.902 (3)	171 (4)
O6W—H6WB···O3^iii^	0.849 (18)	2.117 (19)	2.949 (3)	167 (4)

Symmetry codes: (i) −*x*+1, −*y*+1, −*z*; (ii) *x*, *y*−1, *z*; (iii) *x*+1, *y*, *z*.
